# Stage-Specific Effect of Inositol Hexaphosphate on Cancer Stem Cell Pool during Growth and Progression of Prostate Tumorigenesis in TRAMP Model

**DOI:** 10.3390/cancers14174204

**Published:** 2022-08-30

**Authors:** Komal Raina, Kushal Kandhari, Anil K. Jain, Kameswaran Ravichandran, Paul Maroni, Chapla Agarwal, Rajesh Agarwal

**Affiliations:** 1 Department of Pharmaceutical Sciences, Skaggs School of Pharmacy and Pharmaceutical Sciences, University of Colorado Anschutz Medical Campus, Aurora, CO 80045, USA; 2 Department of Pharmaceutical Sciences, South Dakota State University, Brookings, SD 57007, USA; 3 Department of Surgery, Division of Urology, University of Colorado Anschutz Medical Campus, Aurora, CO 80045, USA; 4 University of Colorado Cancer Center, University of Colorado Anschutz Medical Campus, Aurora, CO 80045, USA

**Keywords:** inositol hexaphosphate, prostate cancer, chemoprevention, cancer stem cells, TRAMP

## Abstract

**Simple Summary:**

The stage of a tumor during cancer intervention is the most crucial factor that determines the treatment regimen. Several bioactive natural compounds have shown potential to inhibit prostate cancer growth and progression; however, there is a dearth of studies that explore their efficacy at different stages of tumorigenesis. This knowledge gap prevents researchers from fully exploiting the anti-cancer potential of these beneficial compounds. Accordingly, our present study focused on explicating the ‘stage-specific’ efficacy of the bioactive food component ‘inositol hexaphosphate (IP6, phytic acid)’ against PCa initiation, growth, and progression in the transgenic adenocarcinoma of the mouse prostate TRAMP model. Results indicated that IP6 feeding during initial stages of cancer development prevents progression of prostatic intraepithelial neoplasia lesions to adenocarcinoma, and IP6 feeding during late stage of the disease reduces tumor growth and prevents its progression to advanced stage of the disease. Thus, IP6 intervention is beneficial during all stages of prostate tumorigenesis.

**Abstract:**

Herein, we assessed the stage-specific efficacy of inositol hexaphosphate (IP6, phytic acid), a bioactive food component, on prostate cancer (PCa) growth and progression in a transgenic mouse model of prostate cancer (TRAMP). Starting at 4, 12, 20, and 30 weeks of age, male TRAMP mice were fed either regular drinking water or 2% IP6 in water for ~8–15 weeks. Pathological assessments at study endpoint indicated that tumor grade is arrested at earlier stages by IP6 treatment; IP6 also prevented progression to more advanced forms of the disease (~55–70% decrease in moderately and poorly differentiated adenocarcinoma incidence was observed in advanced stage TRAMP cohorts). Next, we determined whether the protective effects of IP6 are mediated via its effect on the expansion of the cancer stem cells (CSCs) pool; results indicated that the anti-PCa effects of IP6 are associated with its potential to eradicate the PCa CSC pool in TRAMP prostate tumors. Furthermore, in vitro assays corroborated the above findings as IP6 decreased the % of floating PC-3 prostaspheres (self-renewal of CSCs) by ~90%. Together, these findings suggest the multifaceted chemopreventive-translational potential of IP6 intervention in suppressing the growth and progression of PCa and controlling this malignancy at an early stage.

## 1. Introduction

American Cancer Society data for the year 2022 estimates prostate cancer (PCa) as the most common cancer (~268,000 cases) and the second leading cause of cancer death (~34,000 deaths) in American men [1]. On the global front, with more than 1.4 million cases, PCa is the fourth most commonly diagnosed cancer worldwide [2]. Though localized PCa has a long-term survival rate, on contrary, the metastatic PCa is still largely incurable and the principal cause of PCa related deaths [3]. Improvement in prostate-specific antigen (PSA) screening and advances in clinical practices have helped reduce PCa-associated mortality significantly in the past few decades. However, the incidence rates of localized and metastatic prostate cancer are rising and are expected to increase further in the next decade [4]. Epidemiological studies have also demonstrated significant disparities in PCa incidence worldwide. The disparity in PCa incidence has been attributed to various factors including the variations in the PSA screening, diagnostic practices, ethnicity, family/genetic history, and lifestyle [4,5]. One of the important lifestyle variables other than sedentary factor that has been suggested to be one of the possible reasons for the disparity is the dietary differences between different regions of the world [4,6].

The failure of traditionally used therapies to stem the rising incidence of PCa has compelled researchers to shift their focus to preventive intervention by dietary agents [7,8,9,10]. Inositol hexaphosphate (IP6), also known as phytic acid, is a bioactive food component present in most cereals, legumes, nuts, oilseeds, and soybean [11]. Numerous studies have identified the anti-cancer potential of IP6 against breast, colon, pancreatic, oral, and skin cancer [11,12]; also, several research groups including ours have demonstrated the anti-PCa potential of IP6 in various pre-clinical in vitro and in vivo models [11,12,13,14,15,16,17,18,19,20]. One of our previous studies reported the chemopreventive efficacy of IP6 against prostate cancer growth and progression in the transgenic adenocarcinoma of the mouse prostate TRAMP model [19]. Another study from our group elucidated the molecular mechanism of IP6-induced inhibition of PCa tumor growth, vascularity, and metabolism in TRAMP mice [20]. However, one limitation of these studies was that the chemopreventive intervention started very early and continued throughout the experiment, making it difficult to assess the clinical relevance of IP6 feeding on different tumor stages of PCa. Studies have demonstrated that the ability of dietary factors to prevent cancer is stage-dependent and thus exploring the stage-specific effects may provide insight into the uncharted potential and the associated underlying mechanisms of such dietary compounds [21]. In accordance, our present study focused on explicating the “stage-specific” efficacy of IP6 feeding against PCa initiation, growth, and progression.

## 2. Materials and Methods

### 2.1. Cell Culture and Reagents

PC-3 human prostate carcinoma cells and THP-1 monocytic cells were procured from American Type Culture Collection (ATCC) (Manassas, VA, USA). Both PC-3 and THP-1 cells were routinely cultured in RPMI-1640 medium with 10% fetal bovine serum (FBS) and 1% Penicillin-Streptomycin solution under standard culture conditions (37 °C, 95% humidified air, and 5% CO_2_). THP-1 monocytes were differentiated into macrophages by 24 h exposure to 150 nM phorbol 12-myristate (PMA, #P8139 from Sigma, St. Louis, MO, USA). All cell culture reagents were procured from Gibco, Thermo Fisher Scientific (Waltham, MA, USA) unless otherwise noted. IP6 (#8810 as phytic acid sodium salt hydrate from rice, quality level M-100 from Sigma, St. Louis, MO, USA) was dissolved in water to prepare a 200 mM stock solution (for cell culture use), and pH was adjusted to 7.5. Antibody for PCNA (#M0879) was from DakoCytomation (Glostrup, Denmark). Dead End Colorimetric TUNEL System (#G7130) was purchased from Promega (Madison, WI, USA). Antibodies for VEGF (#ab46154), GLUT4 (#ab654), Sox-2 (#ab97959), and Oct-4 (#ab184665) were from Abcam (Cambridge, MA, USA). Antibody for iNOS (#NB300605) was from Novus Biologicals (Centennial, CO, USA). Antibodies for PECAM-1/CD-31 (#sc-1506) and CXCR3 (#sc-137140) were purchased from Santa Cruz Biotechnology (Dallas, TX, USA). Antibody for Shh (#ARP44235_P050) was from Aviva System Biology (San Diego, CA, USA) and antibody for cleaved Notch-1 (#4147S) was from Cell Signaling Technology (Danvers, MA, USA).

### 2.2. Animals, Treatment, and Necropsy

**Figure 1:**
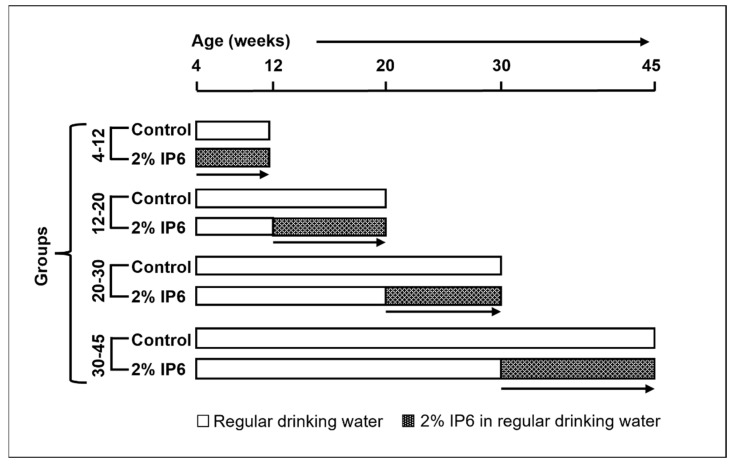
Study design to assess the effect of feeding IP6 during different stages of prostate cancer growth and progression in TRAMP mice. Starting at 4, 12, 20, and 30 weeks of age, male TRAMP mice were fed either regular drinking water or 2% IP6 in water, and then sacrificed at age 12, 20, 30, and 45 weeks respectively. Depending upon the feeding period, the different groups are referred to as 4–12, 12–20, 20–30, and 30–45 week groups, respectively.

TRAMP male mice (4 weeks old) routinely obtained by breeding heterozygous TRAMP (C57BL/6) females with non-transgenic C57BL/6 breeder males were used for this study. Housing and care of the animals were as per the guidelines established by the University of Colorado, Anschutz Medical Campus animal house facility. All the animal protocols were approved by the Institutional Animal Care and Use Committee (IACUC). The mice were randomly distributed into positive control and treatment groups. Male TRAMP mice starting at 4, 12, 20, and 30 weeks of age were fed with regular drinking water (positive control group) or 2% *w/v* IP6 in regular drinking water for ~8–15 weeks as detailed previously [19,20]. Hereafter, different groups depending on their study periods are referred to as 4–12, 12–20, 20–30, and 30–45 week groups, respectively (Figure 1). Number of mice per group: [4–12 weeks: TRAMP controls (n = 11), IP6-fed (n = 13); 12–20 weeks: TRAMP controls (n = 17), IP6-fed (n = 16); 20–30 weeks: TRAMP controls (n = 14), IP6-fed (n = 14); 30–45 weeks: TRAMP controls (n = 11), IP6-fed (n = 12)] and TRAMP negative [untreated WT controls (n = 5) per study group].

At the end of each time point, mice were sacrificed by CO_2_ asphyxiation followed by exsanguination. Lower urogenital tract (LUT) including bladder, seminal vesicles, and prostate were removed *en-bloc*. The prostate gland was harvested and microdissected. Gross pathology of animals, including any evidence of edema, unusual appearance, and abnormal size of any non-target organs, was also noted. All tissues were partly flash-frozen in liquid nitrogen and partly formalin-fixed for further analyses.

### 2.3. Histopathological and Immunohistochemical Analysis

Formalin-fixed tissues were processed as described previously [22]. Histopathological analysis of dorsolateral prostate was done using hematoxylin and eosin (H&E) stained tissues as detailed previously [22]. Given that in the TRAMP model the pathological changes associated with PCa are more evident in the dorsolateral lobes [23], our study assessments focused on these lobes only. For immunohistochemical (IHC) analysis, routine staining technique using 3, 3′-diaminobenzidine (DAB) was employed [24]. Brown-stained cells were counted as positive cells (among total number of cells) and plotted as % positive cells; these were counted in five randomly selected fields at ×400 magnification. Immunoreactivity (intensity of brown staining was represented by arbitrary values) was noted as 0 (no staining), +1 (weak intensity), +2 (moderate intensity), +3 (strong intensity), and +4 (very strong intensity) [25]. Immunopositive area was assessed as the proportion area of prostate which is positive for expression and assigned arbitrary scores as 0 (<5% positive area), +1 (5–25% positive area), +2 (26–50% positive area), +3 (51–75% positive area), and +4 (>75% positive area).

### 2.4. Immunofluorescence Assay

Formalin-fixed dorsolateral prostate tissues were deparaffinized and routinely processed for immunofluorescence assay as described previously [18]. Antibodies used were CD44 (#sc-9960) from Santa Cruz Biotechnology (Dallas, TX, USA) and BMI-1 (#ab38295) from Abcam (Cambridge, MA, USA). The secondary antibodies Goat anti-rabbit IgG Alexa fluor 488 (#A-11008) and Goat anti-mouse IgG Alexa fluor 647 (#A-21236) secondary antibodies were from Invitrogen, Thermo Fisher Scientific (Waltham, MA, USA). Sections were mounted using vectashield antifade mounting medium with 4′,6-diamidino-2-phenylindole (DAPI) (#H-1800) from Vector Laboratories (Burlingame, CA, USA). A Nikon D-Eclipse C1 confocal microscope (Nikon) was used for imaging. All images were taken at ×600; immunofluorescence images were evaluated using QuPath analysis software (Univ. of Edinburgh, Edinburgh, UK, Version 0.3.2).

### 2.5. In Vitro Prostasphere-Formation Assay

FACS sorted CD44^+^ -α2β1^high^ subpopulation of human PCa PC-3 cell line was used for the prostaspheres formation assay in serum-free DMEM/F12 media supplemented with 20 ng/mL rhEGF, 10 ng/mL rhFGF-b, 2% B27, and 1% N2 supplement as described previously [26]. The sorted single cell suspension was plated in 6-well ultra-low attachment plates (Corning) at a density of 3000 cells/well as described previously [26]. Cells were treated with 2 mM IP6, and at the end of the experiment (after 10 days) spheres were examined for number count. To determine the impact of immune cells such as macrophages on prostasphere formation, conditioned media of human macrophage cell line [(PMA differentiated THP-1 monocytes) without or with treatment with 2 mM IP6 for 12 h (followed by drug washout and exposure to serum-free media for 48 h to collect macrophage-conditioned media)] was used in the prostasphere formation assay as above. 

### 2.6. Statistical and Microscopic Analyses

Sigma Stat software (version 3.5, Jandel Scientific Software, San Jose, CA, USA) and GraphPad Prism (version 8.4, GraphPad Software Inc., San Diego, CA, USA) were used for statistical analyses. Incidence of PIN and adenocarcinoma lesions was compared using Fisher’s Exact test and unpaired two-tailed Student’s *t*-test was used for all other data. Quantitative data in the figures are presented as mean ± SEM; *p* ≤ 0.05 was considered statistically significant. As relevant to the study, data were analyzed either intra-group (between age-matched TRAMP positive control and IP6-fed mice) or between WT controls and TRAMP positive controls. Carl Zeiss Axioscope 2 microscope (Carl Zeiss, Inc. Jena, Germany) and attached AxioCam MrC5 camera were employed for all microscopic analyses and capturing of photomicrographs.

## 3. Results

### 3.1. Stage-Specific Effect of IP6 Feeding on Pathological Changes in TRAMP Prostate

**Figure 2:**
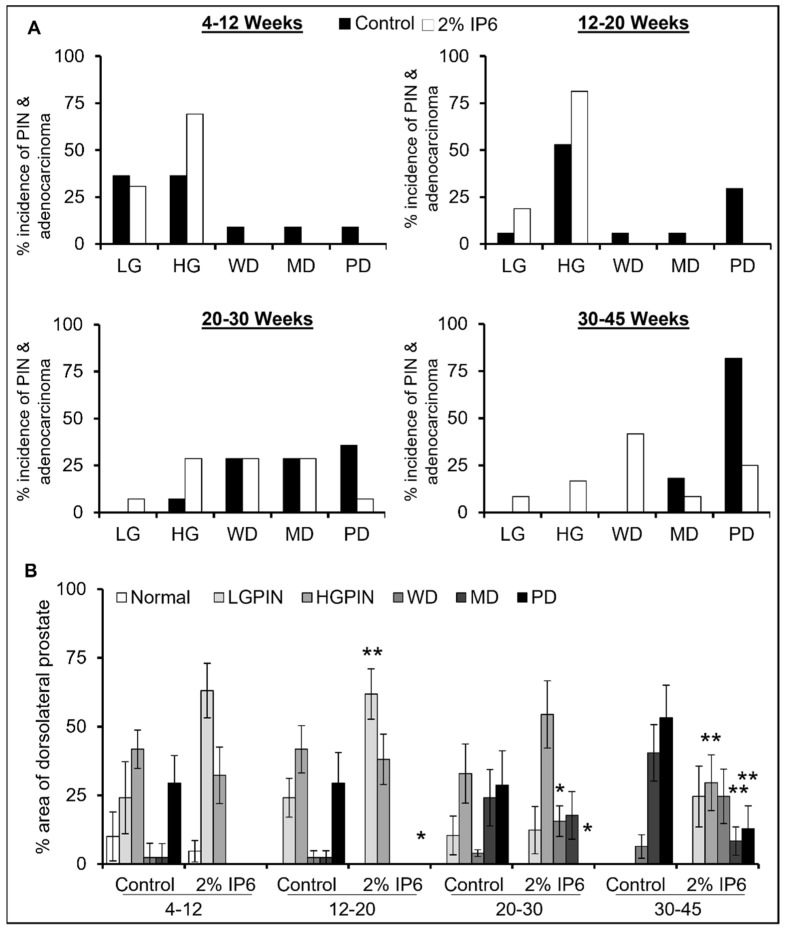
Stage-specific effect of IP6 feeding on pathological changes in dorsolateral prostate of TRAMP mice. (**A**) % incidence of normal, pre-neoplastic, and adenocarcinoma lesions in dorsolateral prostate tissues of TRAMP control and IP6-fed mice. (**B**) % area of dorsolateral prostate lobe displaying normal, pre-neoplastic, and adenocarcinoma lesions in TRAMP control and IP6-fed mice. LGPIN, low-grade prostatic intraepithelial neoplasia; HGPIN, high-grade prostatic intraepithelial neoplasia; WD, well-differentiated (adenocarcinoma); MD, moderately differentiated (adenocarcinoma); PD, poorly differentiated (adenocarcinoma). Quantified data are represented as columns (mean for each group); bars represent SEM. ** *p* ≤ 0.01, and * *p* ≤ 0.05.

There was no significant difference in diet consumption or body weight gain between the TRAMP controls and IP6-fed mice (data not shown); though IP6-fed mice showed lower LUT weight compared to TRAMP controls, the differences were not statistically significant (Supplementary Figure S1). H&E-stained dorsolateral prostate tissue sections were microscopically evaluated and tissues were pathologically classified as described previously [10]. As shown in Figure 2A,B, there was a significant difference between the incidence of PIN and adenocarcinoma lesions between the TRAMP control and IP6-fed groups. Specifically, in the 4–12 weeks control group, ~27% of mice developed adenocarcinoma [~9% incidence each of well-differentiated (WD), moderately differentiated (MD), and poorly differentiated (PD)], whereas no mice in the IP6-fed group developed adenocarcinoma and instead showed ~30% incidence of low-grade PIN and ~70% incidence of high-grade PIN (Figure 2A, *left panel*). A similar effect was seen in the 12–20 weeks group, where ~40% of control mice developed adenocarcinoma and no mice in the IP6-fed group developed adenocarcinoma but only PIN (~19% incidence of low-grade PIN and ~81% incidence of high-grade PIN) (Figure 2A, *right panel*). In the 20–30 weeks group, the IP6-fed group did show incidence of low-grade PIN while there was no incidence of low-grade PIN lesions in control mice. On the other hand, there was a higher incidence of high-grade PIN lesions and a concomitant decrease in PD adenocarcinoma in IP6-fed mice compared to the controls (Figure 2B, *left panel*). In the 30–45 weeks group, all mice in the control group developed high-grade adenocarcinoma (~18% incidence of MD and ~81% incidence of PD), while IP6-fed mice displayed a higher incidence of PIN lesions and WD tumors; the incidence of MD and PD adenocarcinoma was also significantly decreased in the IP6-fed group (Figure 2B, *right panel*). Thus, in the 30–45 weeks cohort, there was an increase in the incidence of differentiated tumors in IP6-fed groups compared with TRAMP controls and a concomitant decrease in the incidence of more advanced tumors (Supplementary Figure S2). 

Furthermore, there was a significant difference in the percentage area of dorsolateral prostate covered by PIN lesions and aggressive adenocarcinoma lesions between the TRAMP control and IP6-fed mice. As can be seen in Figure 2B, the incidence of invasive adenocarcinoma increased as a function of age in the TRAMP controls. The area of dorsolateral prostate covered by adenocarcinoma was highest in the 30–45 weeks TRAMP controls; however, in IP6-treated groups, the majority of the area was covered with non-invasive lesions (LGPIN and HGPIN). Also, in this cohort of IP6-fed mice (30–45 weeks group), there was a significant decrease in the area of MD (~79% decrease, *p* ≤ 0.01) and PD lesions (~76% decrease, *p* ≤ 0.01). 

**Figure 3:**
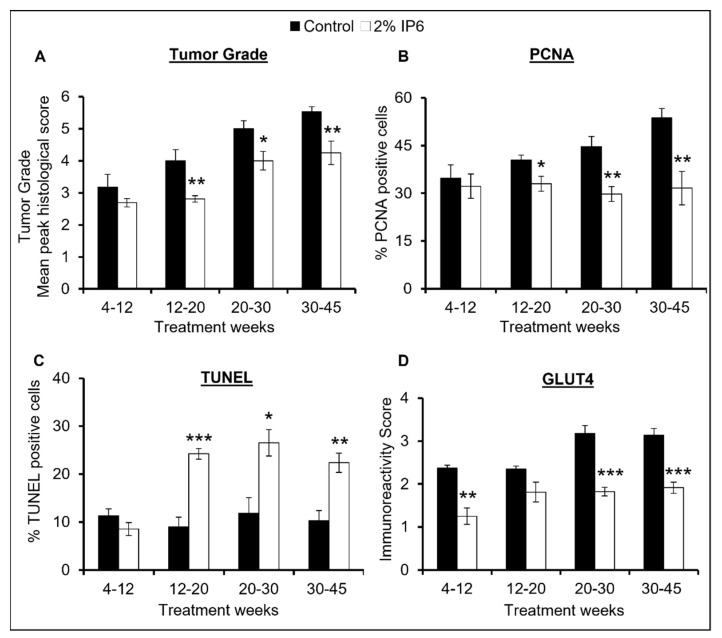
Stage-specific effect of IP6 feeding on tumor grade, proliferation, and apoptosis in dorsolateral prostate of TRAMP mice. Effect on (**A**) Tumor grade, (**B**) PCNA-proliferative index, (**C**) TUNEL, and (**D**) GLUT-4 (glucose transporter) in TRAMP control and IP6-fed mice. DAB, 3,3′-diaminobenzidine; PCNA, proliferating cell nuclear antigen; TUNEL, terminal deoxynucleotidyl transferase dUTP nick end labeling. Quantified data are represented as mean ± SEM. *** *p* ≤ 0.001, ** *p* ≤ 0.01, and * *p* ≤ 0.05.

Next, the severity of dorsolateral prostate lesions was determined by grading the tissues for mean peak histologic score as previously described [16]. As seen in Figure 3A, TRAMP prostates had a mean peak score of ~3.8 (4–12 weeks), ~4.0 (12–20 weeks), ~5.0 (20–30 weeks), and ~5.5 (30–45 weeks) indicating a considerable increase in tumor grade as a function of age. Alternatively, IP6-treated group showed much less severe tumor grade scores in comparison to the control groups. The mean peak score of the IP6-fed group was ~2.7 (4–12 weeks), ~2.8 (12–20 weeks), ~4.0 (20–30 weeks), and ~4.3 (30–45 weeks). This corresponds to a decrease of ~15% (4–12 weeks), ~30% (12–20 weeks, *p* ≤ 0.01), ~20% (20–30 weeks, *p* ≤ 0.05), and ~23% (30–45 weeks, *p* ≤ 0.01), indicating that IP6 feeding during different stages of tumorigenesis also decreases the severity of prostatic lesions in TRAMP mice. Altogether, these results suggest that IP6-feeding is effective in restricting the progression of both pre-malignant neoplastic lesions as well as differentiated tumors to more aggressive forms of adenocarcinoma in the TRAMP prostate.

### 3.2. Stage-Specific Effect of IP6 Feeding on Proliferation Index and Apoptosis in TRAMP Prostate

To investigate the effect of IP6 feeding on the proliferative index in prostate tissues, immunostaining for proliferating cell nuclear antigen (PCNA) was performed on dorsolateral prostate tissues. Quantitative microscopic analyses of the stained tissues revealed that PCNA-positive cell percentage in TRAMP controls increased in an age-dependent manner. Specifically, the percentage of PCNA positive cells in the TRAMP mice was ~35% (4–12 weeks group), ~41% (12–20 weeks group), ~45% (20–30 weeks group), and ~54% (30–45 weeks group), whereas the percent PCNA positive cells in the IP6-fed group was ~32% (4–12 weeks group), ~33% (12–20 weeks group), ~30% (20–30 weeks group), and ~32% (30–45 weeks group). This corresponds to a significant decrease of ~20% (12–20 weeks group, *p* ≤ 0.05), ~33% (20–30 weeks group, *p* ≤ 0.01), and ~41% (30–45 weeks group, *p* ≤ 0.01) in the IP6-fed mice, implying towards a more significant effect of IP6 feeding on the proliferative index in the advanced stages of PCa (Figure 3B).

Next, the apoptotic effect of IP6 feeding in dorsolateral prostate tissues of the TRAMP mice was assessed. Terminal deoxynucleotidyl transferase biotin-dUTP nick-end labeling (TUNEL) assay on the tissue samples was performed and microscopy-based examination of tissues demonstrated an increased number of apoptotic cells in the IP6-fed groups (Figure 3C). Specifically, IP6 feeding increased the number of TUNEL positive (apoptotic) cells by ~2.6 fold (12–20 weeks group, *p* ≤ 0.001), ~2.3 fold (20–30 weeks group, *p* ≤ 0.05), and ~2.2 fold (30–45 weeks group, *p* ≤ 0.01) compared to TRAMP controls. 

Thereafter, we assessed the effect of IP6 feeding on the expression of glucose transporter GLUT4 in dorsolateral prostate tissue of TRAMP mice. Studies in the past have established that PCa cells overexpress GLUT4 which plays a vital role in fulfilling the energy needs of highly proliferative PCa cells [27]. Aberrant glucose uptake in cancer cells is also known to help in PCa growth and progression [28]. IHC analysis for GLUT4 in prostate tissues revealed that the expression of GLUT4 increased as a function of age in the TRAMP controls and IP6 feeding was able to substantially decrease the expression of GLUT4 (Figure 3D). Specifically, a decrease of ~47% (4–12 weeks group, *p* ≤ 0.01), ~23% (12–20 weeks group), ~43% (20–30 weeks group, *p* ≤ 0.001), and ~39% (30–45 weeks group, *p* ≤ 0.001) in the expression of GLUT4 was observed in the IP6-fed mice compared to TRAMP controls. Overall, these observations suggest that IP6 feeding inhibits the proliferative potential and induces apoptosis in the TRAMP mice prostate cells and that the effect is more evident in the later stages of tumorigenesis.

### 3.3. Stage-Specific Effect of IP6 Feeding on Angiogenesis and Associated Regulatory Molecules in TRAMP Prostate

**Figure 4:**
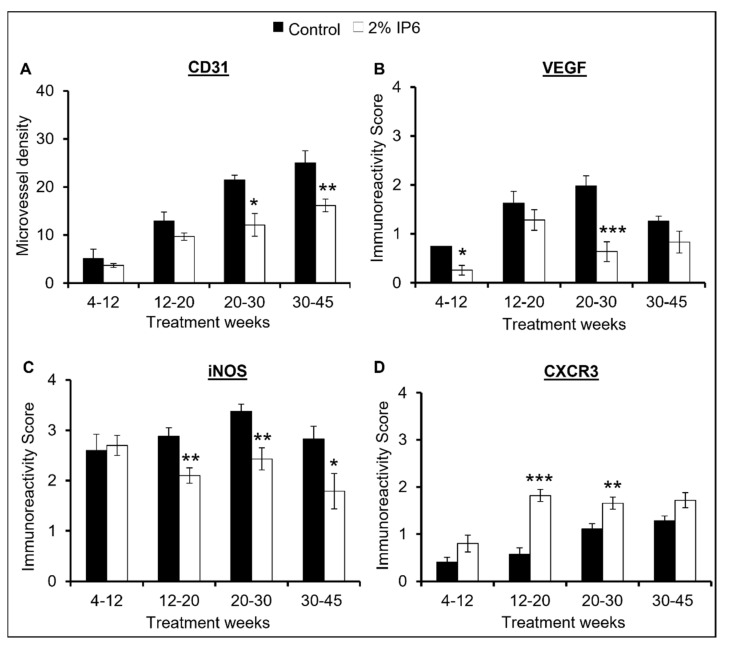
Stage-specific effect of IP6 feeding on angiogenic pathway in dorsolateral prostate of TRAMP mice. Effect on (**A**) microvessel density (MVD) as inferred by expression of PECAM-1/CD-31. MVD was determined by calculating the number of positive foci counted under ×400 magnifications in five selected areas in each section. Effect on (**B**) VEGF, (**C**) iNOS, (**D**) CXCR3 expression in TRAMP mice prostate and IP6-fed mice as determined by IHC. Quantified data are represented as mean ± SEM. *** *p* ≤ 0.001, ** *p* ≤ 0.01, and * *p* ≤ 0.05.

CD-31: IHC analysis of the dorsolateral prostate tissues for the endothelial cell marker, platelet endothelial cell adhesion molecule-1/cluster of differentiation 31 (PECAM-1 or CD31), showed that the expression of CD-31 increased as a function of age in the TRAMP mice. However, IP6-fed mice showed an overall decrease in the expression of CD-31, especially in the later age groups, suggesting a potent efficacy of IP6 to reduce de novo angiogenesis in the TRAMP prostate. Specifically, CD-31 expression decreased by ~28% (4–12 weeks group), ~23% (12–20 weeks group), ~54% (20–30 weeks group, *p* < 0.05), and 36% (30–45 weeks group, *p* ≤ 0.01) in the IP6-fed mice compared to TRAMP controls (Figure 4A). 

VEGF: The effect of IP6 feeding on the expression of VEGF, an angiogenesis regulator, was also analyzed in prostate tissues. Similar to the results of CD-31, IHC analysis demonstrated that IP6 feeding substantially decreased the expression of VEGF. There was a reduction of ~65% (4–12 weeks group, *p* ≤ 0.05), ~21% (12–20 weeks group), ~68% (20–30 weeks group, *p* ≤ 0.001), and ~34% (30–45 weeks group) in VEGF expression in IP6-fed mice compared to controls (Figure 4B). Overall, this result in combination with the CD-31 data corroborates our histopathological analysis and suggests that IP6 feeding at different stages has the potential to impact angiogenesis in TRAMP prostate.

iNOS: Inducible nitric oxide synthase (iNOS) has been shown to play a role in PCa progression by favoring proliferation as well as angiogenesis [29,30], as such we analyzed the expression of iNOS in the prostate tissues from control and IP6-fed TRAMP mice in all age groups. IHC analysis demonstrated that IP6 treatment significantly decreased the expression of iNOS in the later stages of tumorigenesis. Specifically, iNOS expression was decreased by ~27% (12–20 weeks group, *p* ≤ 0.01), ~28% (20–30 weeks group, *p* ≤ 0.01), and ~37% (30–45 weeks group, *p* ≤ 0.05) in the IP6-fed mice compared to the TRAMP controls (Figure 4C).

CXCR3: There is accumulating evidence that CXCR3 is a vital angiostatic receptor for various CXC chemokines such as CXCL9, CXCL10, and CXCL11 [31,32,33]. CXCL10-induced CXCR3 expression has been associated with reduced cell proliferation and decreased PSA levels in PCa cells [34]. Therefore, we analyzed the expression of CXCR3 in TRAMP dorsolateral prostate tissues by IHC and found that IP6 treatment was able to increase the expression of CXCR3 in all age groups. There was an increase of ~2.0 fold (4–12 weeks group), ~3.1 fold (12–20 weeks group, *p* ≤ 0.001), ~1.5 fold (20–30 weeks group, *p* ≤ 0.01), and ~1.3 fold (30–45 weeks group) in the IP6-fed mice compared to TRAMP controls (Figure 4D). 

### 3.4. Stage-Specific Effect of IP6 Feeding on the Expansion of Cancer Stem Cells (CSCs) Pool in TRAMP Prostate

Cancer stem cells (CSC) endowed with tumor-initiating and self-renewal capacities have been recognized as the driving force for tumor initiation and progression to advanced stages in different epithelial cancers including PCa [35,36,37,38]. CD44 is a cell-surface protein involved in cell adhesion, tumor invasion, and metastasis and its high expression is also recognized as a phenotypic marker for tumor-initiating cells (TICs) [39]. BMI-1 is responsible for cell proliferation, cell motility, self-renewal, and therapy resistance in PCa cells, and is also recognized to play a vital role in self-renewal of TICs [40]. To determine whether the protective effects of IP6 are mediated via the effect on the expansion of the TICs/CSCs pool, we analyzed the TICs pool (for dual expression of CD44 and BMI-1) as a function of tumor aggressiveness (with or without IP6 treatment).

**Figure 5:**
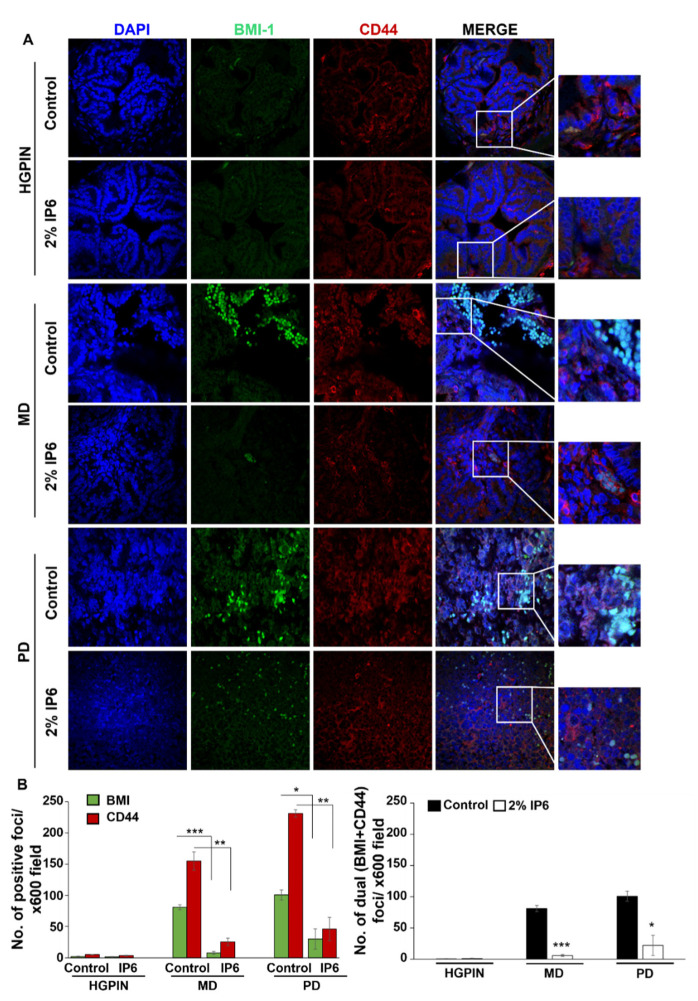
Stage-specific effect of IP6 feeding on the expansion of cancer stem cells (CSCs) pool in dorsolateral prostate of TRAMP mice. Immunofluorescence (IF) studies to determine the correlation and dual stained (BMI-1 and CD44 expression) tumor initiating cells (TICs/CSCs) pool in different pathological lesions of dorsolateral prostate of TRAMP controls and IP6-fed groups. Tissues were dual-stained for BMI-1 (green) and CD44 (red) expression. Nuclear staining was done with DAPI (blue). (**A**) Representative pictographs are depicted at x600 magnification and insets represent digital magnifications. (**B**) BMI-1 and CD44 positive foci was quantified using QuPATH analysis software. Quantified data are represented as mean ± SEM. *** *p* ≤ 0.001, ** *p* ≤ 0.01, and * *p* ≤ 0.05.

Results revealed that both CD44 and BMI-1 had minimal expression in PIN stages and their expression increased with tumorigenesis, i.e., a strong expression was observed in MD and PD stages in TRAMP controls (Figure 5A,B). On the other hand, IP6 treatment was able to induce a significant decrease in the expression of CD44 (*p* ≤ 0.01, both MD and PD stages) and BMI-1 (*p* ≤ 0.001, MD stage; *p* ≤ 0.05, PD stage). Notably, in the IP6-fed groups, the cells that dual stained for CD44/BMI were significantly decreased (*p* ≤ 0.001, MD stage; *p* ≤ 0.05, PD stage) compared to that present in TRAMP controls (Figure 5B, *right panel*) indicating the possibility of IP6 decreasing the TICs/CSCs pool.

### 3.5. In Vitro Effect of IP6 Treatment on Prostasphere Formation

**Figure 6:**
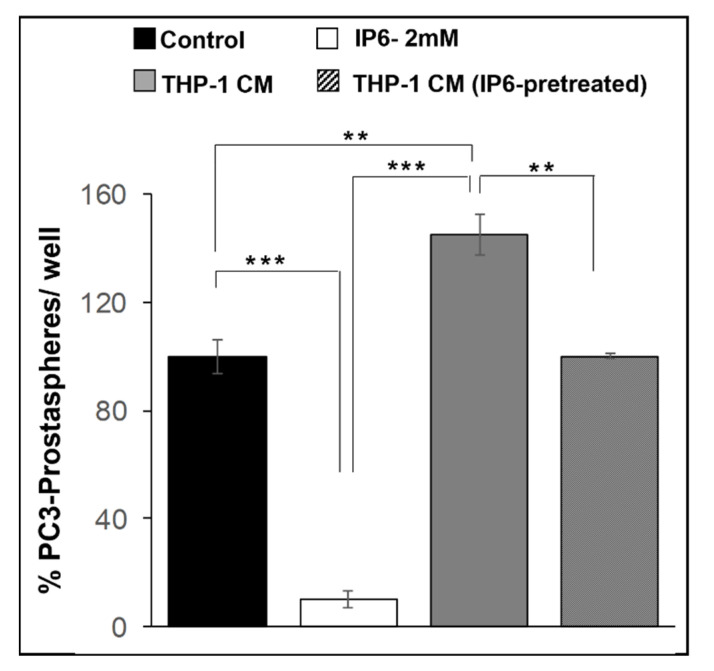
Effect of IP6 treatment on TICs/CSCs enriched prostaspheres in human prostate cancer PC-3 cancer cells. Effect of 2 mM IP6 treatment on TICs/CSCs (CD44^+^-α2β1^high^) enriched prostaspheres formation, and effect on prostaspheres formation in the presence of macrophage THP-1 conditioned media and macrophage THP-1 conditioned media (pre-treated with IP6). Quantified data are represented as mean ± SEM. *** *p* ≤ 0.001 and ** *p* ≤ 0.01.

Next, to corroborate the above findings on the potential of IP6 feeding to modulate the expansion of TICs pool and to determine the effect on the self-renewal capacity of prostate TICs/CSCs, we performed an in vitro prostasphere formation assay employing TICs/CSCs enriched (CD44^+^ -α2β1^high^) PC-3 cells sub-population. Importantly, the % of floating spheroids (prostaspheres) generated in the presence of 2 mM IP6 was decreased by ~90% (*p* ≤ 0.001) compared to control. Additionally, to account for other microenvironment triggers (such as inflammatory components) that can stimulate self-renewal capacity, the % of prostaspheres generated in the presence of macrophage THP-1 conditioned media (with and without IP6 pre-treatment) was determined. The results indicated that in vitro prostasphere assay performed with THP-1 conditioned media caused a ~1.5-fold increase (*p* ≤ 0.01) in PC-3 prostaspheres formation compared to regular non-conditioned assay media. Notably, THP-1 conditioned media collected after pre-treatment of macrophages with IP6 lost its stimulating effect on self-renewal of PC-3 TICs/CSCs and was able to decrease the % prostasphere formation by ~45% (*p* ≤ 0.01). Since formation of spheroids under specific in vitro conditions is a measure of stemness, it is evident that IP6 has the potential to target the self-renewal of TICs/CSCs in PCa cells (Figure 6).

### 3.6. Stage-Specific Effect of IP6 Feeding on the Expression of CSC-Associated Signaling Molecules and Transcription Factors in TRAMP Prostate

TICs/CSCs highly express stemness-associated regulatory molecules and transcription factors such as Notch1, Shh, Sox-2, and Oct4. These molecules are associated with various signaling pathways that are recognized to be involved in tumor initiation, progression, self-renewal, stemness, neo-angiogenesis, and therapeutic resistance [41].

**Figure 7:**
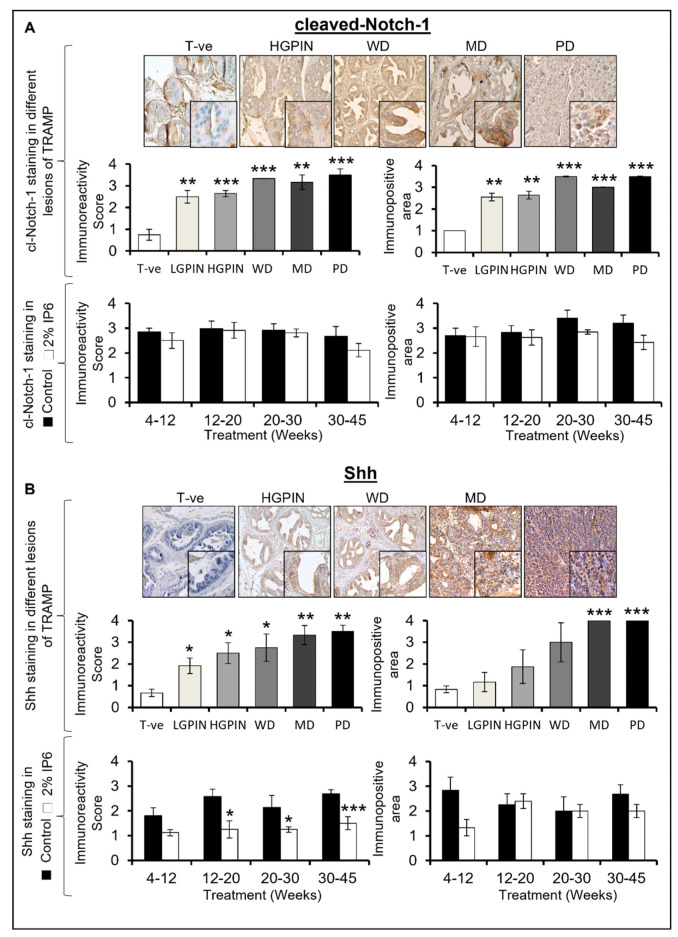
Stage specific effect of IP6 feeding on the expression of CSC-associated signaling molecules in the dorsolateral prostate of TRAMP mice. Pictographs and bar graphs representing the stage-specific expression of CSC-associated signaling molecules (**A**) cleaved-Notch-1, and (**B**) Shh, in WT control (T-ve), TRAMP control, and IP6-fed mice. Representative pictographs are depicted at ×100 magnification and insets represent digital magnifications. Immunoreactivity was scored as 0 (no staining), +1 (weak), +2 (moderate), +3 (strong), and +4 (very strong). The proportion area of prostate (positive for expression) was quantified as  immunopositive area score and assigned arbitrary scores as 0 (<5% area), +1 (5–25% area), +2 (26–50% area), +3 (51–75% area), and +4 (>75% area). Quantified data are represented as mean ± SEM. *** *p* ≤ 0.001, ** *p* ≤ 0.01, and * *p* ≤ 0.05.

Cleaved Notch-1: IHC analysis for cleaved Notch-1 (activated Notch-1) expression revealed that its expression was markedly increased in all stages of PCa, especially in the more aggressive stages; however, there was no difference between low-grade and high-grade PIN stage expression, and the difference in cleaved Notch-1 expression was almost similar between WD, MD, and PD stages. Specifically, there was an increase of ~3.3 fold (LGPIN, *p* ≤ 0.01), ~3.5 fold (HGPIN, *p* ≤ 0.001), ~4.4 fold (WD, *p* ≤ 0.001), ~4.2 fold (MD, *p* ≤ 0.01), and ~4.6 fold (PD, *p* ≤ 0.001) (Figure 7A, *upper left panel*) in the expression compared to WT controls. Likewise, the proportional area of dorsolateral prostate tissue having cleaved Notch-1 expression (immunopositive area) also increased compared to WT controls. Overall, there was an increase of ~2.5–3.5 fold in the average area of the prostate tissue having cleaved Notch-1 expression (*p* ≤ 0.01) (Figure 7A, *upper right panel*) compared to WT controls. Next, we also assessed the effect of IP6 treatment on the expression and the area of dorsolateral positive for cleaved Notch-1 expression as a function of mice age. Results indicated that IP6-feeding had no significant effect on the cleaved Notch-1 expression nor was there any significant change in the proportion of prostate area positive for cleaved Notch-1 staining (Figure 7A, *lower panels*).

Shh: The expression of Shh increased significantly as a function of lesion stage, with a very strong expression in the adenocarcinoma stages. An increase of ~2.8 fold (LGPIN, *p* ≤ 0.05), ~3.8 fold (HGPIN, *p* ≤ 0.05), ~4.1 fold (WD, *p* ≤ 0.05), ~5.0 fold (MD, *p* ≤ 0.01), and ~5.3 fold (PD, *p* ≤ 0.01) was observed for Shh expression in TRAMP prostates compared to WT controls (Figure 7B, *upper left panel*). The proportion of prostate area positive for Shh staining also increased in TRAMP controls with an increase in the aggressiveness of the tumor; specifically, there was an increase of ~1.3 fold (LGPIN), ~2.2 fold (HGPIN), ~3.6 fold (WD), and ~4.8 fold (for both MD and PD, *p* ≤ 0.001) (Figure 7B, *upper right panel*) compared to WT controls. On the other hand, IP6 treatment was able to significantly decrease the expression of Shh across all the study time points. A decrease of ~38% (4–12 weeks group), ~51% (12–20 weeks group, *p* ≤ 0.05), ~42% (20–30 weeks group, *p* ≤ 0.05), and ~44% (30–45 weeks group, *p* ≤ 0.001) in Shh expression was observed in the IP6-fed groups when compared to TRAMP controls (Figure 7B, *lower left panel*). Though a decrease in the Shh immunopositive area was also noted in the 4–12 and 30–45 weeks group by IP6 feeding, it was not statistically significant (Figure 7B, *lower right panel*).

**Figure 8:**
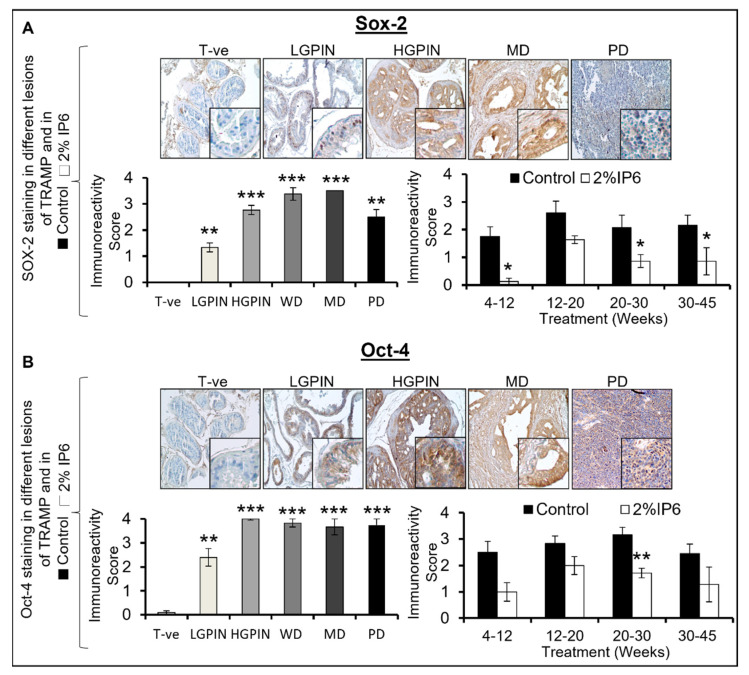
Stage-specific effect of IP6 feeding on the expression of CSC-associated transcription factors in the dorsolateral prostate of TRAMP mice. Pictographs and bar graphs representing the stage-specific expression of CSC-associated transcription factors (**A**) Sox-2, and (**B**) Oct-4, in WT control (Tr-ve), TRAMP control, and IP6-fed mice. Representative pictographs are depicted at ×100 magnification and insets represent digital magnifications. Immunoreactivity was scored as 0 (no staining), +1 (weak), +2 (moderate), +3 (strong), and +4 (very strong). Quantified data are represented as mean ± SEM. *** *p* ≤ 0.001, ** *p* ≤ 0.01, and * *p* ≤ 0.05.

Sox-2: Aberrant expression of Sox-2 can play a vital role in cancer progression by affecting the signaling pathways involved in tumor initiation, cell proliferation, epithelial-to-mesenchymal transition (EMT), migration, invasion, CSC regulation, and resistance to apoptosis and therapy [42]. Accordingly, we assessed the expression pattern of Sox-2 as a function of PCa aggressiveness. The expression of Sox-2 increased with aggressiveness in TRAMP prostate; however, in the PD stages, the expression was lower than in the MD stage. Specifically, Sox-2 expression increased by ~1.3 fold (LGPIN, *p* ≤ 0.01), ~2.7 fold (HGPIN, *p* ≤ 0.001), ~3.4 fold (WD, *p* ≤ 0.001), ~3.5 fold (MD, *p* ≤ 0.001), and ~2.5 fold (PD, *p* ≤ 0.01) (Figure 8A, left panel) in TRAMP prostate compared to WT controls. IHC analysis in the IP6-fed groups revealed that Sox-2 expression was considerably decreased across all age groups. Specifically, the decrease in the immunoreactive score of Sox-2 in the IP6-fed group was ~93% (4–12 weeks group, *p* ≤ 0.05), ~37% (12–20 weeks group), ~58% (20–30 weeks group, *p* ≤ 0.05), and ~60% (30–45 weeks group, *p* ≤ 0.05) (Figure 8A, *right panel*).

Oct-4: Oct-4 is a transcription factor involved in CSC maintenance and other associated oncogenic signaling pathways [43]. IHC analysis showed a marked increase in the expression of Oct-4 in PIN and adenocarcinoma stages; however, the expression in high-grade PIN and in WD, MD, and PD lesions was almost similar. Oct-4 expression increased by ~30 fold (LGPIN, *p* ≤ 0.01), ~50 fold (HGPIN, *p* ≤ 0.001), ~48 fold (WD, *p* ≤ 0.001), ~45 fold (MD, *p* ≤ 0.001), and ~47 fold (PD, *p* ≤ 0.001) (Figure 8B, *left panel*) compared to WT controls. Notably, IP6 treatment decreased the expression of Oct-4 across all age groups. Specifically, IP6 treatment decreased the Oct-4 expression in the TRAMP prostates by ~60% (4–12 weeks group), ~29% (12–20 weeks group), ~46% (20–30 weeks group, *p* ≤ 0.01), and ~48% (30–45 weeks group) (Figure 8B, *right panel*). Collectively, these results suggest that IP6 feeding can restrict the expansion of the TICs/CSC pool in the PCa by downregulating key molecular markers associated with the stemness and self-renewal-associated signaling pathways.

## 4. Discussion

Chemoprevention/intervention using natural non-toxic compounds has emerged as one of the alternate and viable therapies to control cancer prevalence [44]. This approach involves “halting or delaying” cancer at critical stages of carcinogenesis such as tumor initiation, promotion, and progression [45,46]. IP6, a naturally occurring poly-phosphorylated carbohydrate present in many dietary sources with high fiber content such as legumes, has been explored for its anti-cancer efficacy against various cancers [11,12,47]. Several studies have also demonstrated that IP6 does not cause any apparent toxicity in cell culture and animal models of different origins including the PWR-1E normal prostate epithelial cells and TRAMP mouse model [11,12,48].

In the present study, we focused on elucidating the stage-specific efficacy of IP6 against PCa growth and progression in TRAMP mice. IP6 (2% in drinking water) was fed to TRAMP mice at different stages of PCa development and then the efficacy of IP6 was evaluated on PCa growth, progression, angiogenesis, and expansion of the CSC pool. Notably, IP6 feeding to TRAMP mice at an early age restricted the onset of neoplastic characteristics and delayed the tumor growth to advanced stages. As early as 4–12 weeks of treatment regimen, in IP6-fed mice, 100% of prostate tissue was restricted to PIN stages only, whereas TRAMP prostate advanced to more aggressive adenocarcinoma lesions. This trend continued in other study time points groups with IP6-fed groups displaying much less advanced PCa lesions and TRAMP controls displaying an increased incidence of invasive adenocarcinoma lesions. For example, in the 12–20 weeks group, IP6 feeding restricted the tumor growth to PIN lesions only, while in the 20–30 and 30–45 weeks cohorts the incidence of adenocarcinoma lesions was significantly lower in IP6-fed mice compared to the TRAMP controls. This observation was also supported by the moderate decrease in the percent area covered by PIN and adenocarcinoma lesions throughout all the stages of PCa in IP6-fed TRAMP mice. IP6 feeding also reduced the mean peak tumor grades, thus suggesting that IP6 reduces the prostatic tumor lesion severity in TRAMP mice. These observations indicate the clinical potential of IP6 in restricting the growth and progression of PCa at different stages of tumorigenesis. 

Aberrant cellular proliferation and evasion of apoptosis are some of the most important hallmarks of cancer [49]. Several natural compounds and dietary phytochemicals have been studied for their ability to inhibit proliferative ability and induce apoptosis in cancer cells [50]. In previous studies, IP6 was shown to inhibit cellular proliferation and induce apoptosis in PCa cells [16,17,18]. In the present study, IP6 considerably decreased the proliferative index and induced apoptosis in the prostatic tissues of TRAMP mice across all tumor stages. 

Cancer cells exhibit high levels of aerobic glycolysis to meet the needs of aberrantly proliferating cells. In this regard, dysregulated expression of glucose transporters (GLUTs) has been observed in cancer cells [51]. GLUT4, one of the glucose transporters, is highly expressed in PCa cells [28]. Immunohistochemical staining for GLUT4 in TRAMP prostate showed a significant increase in the expression of GLUT4 as a function of mice age. On the contrary, IP6-fed groups showed a significant reduction in the expression of GLUT4 across all stages, especially in the later time points of the study. This observation suggests that IP6 exerts its anticancer efficacy by modulating glucose uptake and transport in different stages of tumorigenesis.

Activation of neo-angiogenesis is one of the early prerequisites for cancer progression to advanced stages [52]. VEGF expression has been inversely associated with survival in PCa cancer patients [53]; studies have also indicated that microvessel density increases as a function of tumor grade in prostate cancer [54]. In this regard, we observed that IP6 treatment was able to decrease the expression of both CD-31 and VEGF substantially when compared to the TRAMP controls in different stages of tumorigenesis. Additionally, IP6 treatment also modulated the expression of iNOS and CXCR3, which are recognized to play critical roles in PCa progression [30,31,32,33]. Overall, these results establish the anti-angiogenic potential of IP6 in stemming the progression of prostate cancer to advanced stages.

PCa is a highly heterogeneous cancer, genetically as well as phenotypically, which makes it difficult to treat using conventional therapies [55]. The TICs/CSCs are a subpopulation of tumor cells that are predominantly recognized as the ones imparting this heterogeneity to PCa [55]. TICs/CSCs are endowed with tumor-initiating potential and can also aid in growth and progression to advanced stages. These cells have distinct biomarkers, and they exhibit high plasticity which allows them to change their phenotypic and functional profile [56] and they can be identified based on the cell surface molecular markers such as CD44 and CD133 [57]. Genetic characterization of TICs/CSCs can be performed by investigating the expression of stemness genes, transcription factors, and associated regulatory molecules such as Oct-4, Sox-2, Shh, BMI-1, and Notch-1 [57,58]. These molecular markers collectively help maintain the TICs/CSC pool, which is vital for increased aggressiveness in cancers. Several studies have established the role of the above-mentioned markers in imparting self-renewal and therapy resistance to PCa cells [35,57,59]. A dual staining assay for CD44 and BMI-1 demonstrated that there was an elevated expression of CD44 and BMI-1 in the TRAMP prostates which was considerably downregulated by IP6 treatment across all tumor stages. Additionally, an in vitro prostasphere corroborated the above inhibitory effects of IP6 on the TICs/CSC pool (even in the presence of stimulatory signals from the immune compartment) suggesting that IP6 inhibitory effects on PCa growth could be partly attributed to its potential to target self-renewal of TICs/CSCs. Furthermore, while TICs/CSCs-associated molecular markers increased with tumorigenesis in the TRAMP prostate, IP6 feeding significantly decreased the expression of these molecules, with the most significant effect on the expression levels of Shh, Sox-2, and Oct-4. These results indicate that the molecular markers associated with the TICs/CSCs pool play a vital role in PCa progression and that IP6 exerts its inhibitory PCa effects by impacting this vital cell pool driving prostate tumorigenesis. Taken together, the efficacy outcomes from different stages of PCa and parallel molecular assessments indicated that IP6 feeding could impact tumor metabolism via interfering in glucose uptake (due to its effect on GLUT-4 expression) which in turn could slow down tumor proliferation early on. Furthermore, during the progression phase of PCa, IP6 feeding, apart from interfering with tumor metabolism, restricted angiogenesis promoting signals which arrested progression to advanced stages of PCa. In addition, the beneficial effects of IP6 against PCa could also be attributed to its negative impact on the CSC pool directly, as well as the tumor promoting signaling originating from the tumor microenvironment, which could benefit in early as well as late phases of tumorigenesis (including tumor recurrence). However, the present study did not investigate the in-depth mechanistic associations which could help establish the upstream and downstream modulators involved in cross-signaling nexus between the pathways modulated by IP6; such studies are warranted in future to delineate the anti-PCa mechanism associated with IP6 intake. 

## 5. Conclusions

Together, these observations are highly significant and for the first time establish the stage-specific efficacy of IP6 feeding during prostate tumorigenesis in TRAMP mice. The present study, in combination with our earlier findings in pre-clinical in vitro and in vivo models including TRAMP mice, implies a strong efficacy of inositol hexaphosphate against all stages of prostate tumorigenesis with scientific rationale and it advocates for future clinical trials in patients with PIN and/or low to high-grade PCa.

## Data Availability

The data presented in this study are available on request from the corresponding author.

## Supplementary Materials

The following supporting information can be downloaded at: https://www.mdpi.com/article/10.3390/cancers14174204/s1, Figure S1: Stage-specific effect of IP6 feeding on the LUT weight of TRAMP mice; Figure S2: Representative pictographs (×100 magnification) of H&E stained dorsolateral prostate tissue from TRAMP control and IP6-fed TRAMP mice in the 30–45 week group.

## Abbreviations

CSCs	Cancer stem cells
DAB	3, 3 -diaminobenzidine
FACS	Fluorescence-activated cell sorting
HGPIN	High-grade prostatic intraepithelial neoplasia
IHC	Immunohistochemistry
LGPIN	Low-grade prostatic intraepithelial neoplasia
LUT	Lower urogenital tract
MD	Moderately differentiated
PCa	Prostate cancer
PCNA	Proliferating cell nuclear antigen
PD	Poorly differentiated
PIN	Prostatic intraepithelial neoplasia
PMA	Phorbol 12-myristate
Shh	Sonic hedgehog
TICs	Tumor-initiating cells
TUNEL	Terminal deoxynucleotidyl transferase-mediated dUTP nick end labeling
WD	Well-differentiated
