# Supplementary material for: Stage-Specific Effect of Inositol Hexaphosphate on Cancer Stem Cell Pool during Growth and Progression of Prostate Tumorigenesis in TRAMP Model
Source: Cancers (Basel). 2022 Aug 30;14(17):4204. doi: 10.3390/cancers14174204 (PMC9455012; doi:10.3390/cancers14174204)
Supplement: Supplementary file 1 [file cancers-14-04204-s001.zip › cancers-1822592-supplementary.pdf]

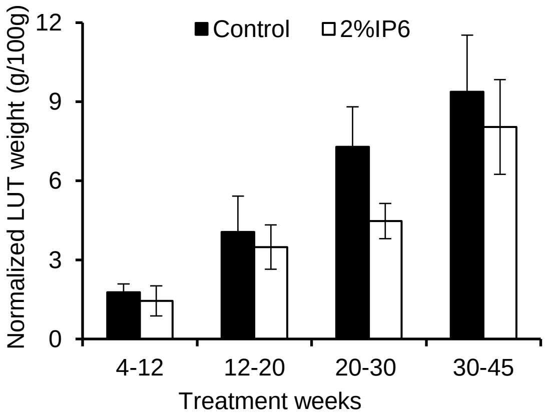

Supplementary Figure S1. Stage-specific effect of IP6 feeding on the LUT weight of TRAMP mice. Quantified data are represented as columns (mean for each group); bars represent mean±SEM. *IP6*, inositol hexaphosphate. LUT, lower urinogenital tract prostate, bladder, and seminal vesicles).

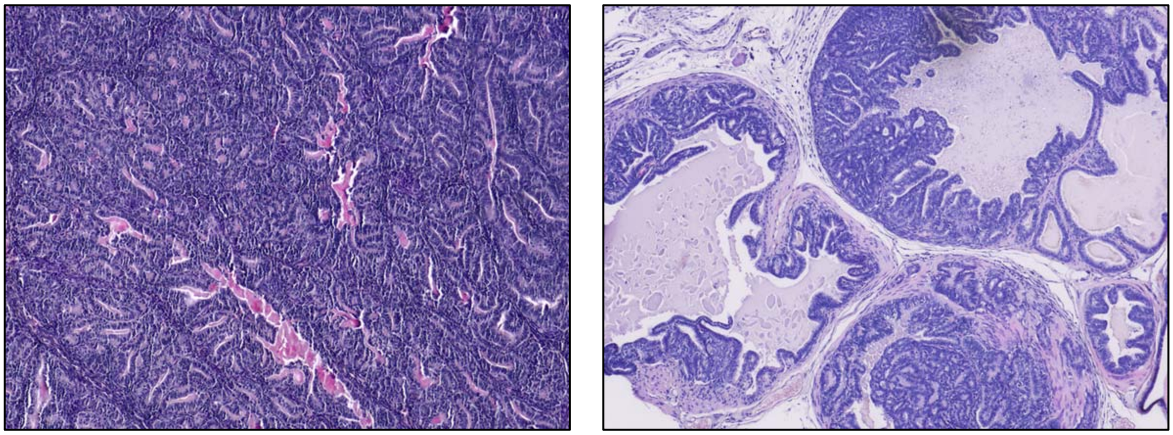

Control **2% IP6**

Supplementary Figure S2. Representative pictographs (x100 magnification) of H&E stained dorsolateral prostate tissue from TRAMP control (left) and IP6-fed TRAMP mice (right) in the 30-45 week group. *IP6*, inositol hexaphosphate.
